# Antidepressive response of inpatients with major depression to adjuvant occupational therapy: a case–control study

**DOI:** 10.1186/s12991-016-0124-0

**Published:** 2017-01-03

**Authors:** Marc-Andreas Edel, Brian Blackwell, Markus Schaub, Barbara Emons, Tanja Fox, Friederike Tornau, Bernward Vieten, Patrik Roser, Ida Sibylle Haussleiter, Georg Juckel

**Affiliations:** 1 Department of Psychiatry, Psychotherapy and Preventive Medicine, LWL University Hospital, Ruhr University Bochum, Alexandrinenstr. 1-3, 44791 Bochum, Germany; 2 Institute of Mental Health, LWL University Hospital Bochum, Bochum, Germany; 3 LWL Hospital Paderborn, Paderborn, Germany

**Keywords:** Adjuvant occupational therapy, Major depression, Antidepressive effects

## Abstract

**Background:**

Despite marked costs and limited evidence regarding effectiveness, occupational therapy (OT) is widely applied in psychiatric settings and financed by health insurance companies in European countries. This pilot study investigated the antidepressive effects of adjuvant OT for patients with major depression in a 6-week inpatient setting, stratified for females and males.

**Methods:**

A total of 114 inpatients with major depression were assigned to either a standard OT group (using basic handcraft) or an active control group that played board games (2 h daily, 5 days a week). HAMD-21 scores were assessed as the primary outcome parameter after 3–6 weeks.

**Results:**

The OT intervention was not superior to “board game” (BG) activities in reducing depressive symptoms. However, significant interaction effects were found in favor of the OT group regarding anxiety measures and other variables. Male participants displayed more significant interaction effects than female participants.

**Conclusions:**

OT as an adjuvant short-term treatment for inpatients with major depression may be more efficacious than game interventions in terms of reducing anxiety and other symptoms, particularly in males.

*Trial registration* The study was registered in the EU Clinical Trials Register as a multicenter trial (EudraCT Number 2009-016463-10; https://www.clinicaltrialsregister.eu/ctr-search/trial/2009-016463-10/DE#A)

However, because of the elaborate setting requirements, the original study design with four centers was transformed into a solution with those two centers facilitating the pertinent resources. Furthermore, “mono-therapy with mirtazapine” was changed into “preferably mono-therapy with any antidepressant drug”.

## Background

Major depression is one of the most common and debilitating mental disorders. It causes enormous individual, social, and economic burden [18]. According to the German Information System of the Federal Health Monitoring, the diagnosis of a major depressive episode is the number one cause for inpatient treatment in German psychiatric hospitals based on consistent data collected in the years 2000–2010 [5].

In several European countries, occupational therapy (OT) is known as *Ergotherapie* (from the Greek *ergon* = work, exercise). In German-speaking countries, it is a traditional treatment that is most frequently applied in combination with other treatments, such as pharmacotherapy [14].

Occupational therapy is based on the positive relation between meaningful occupation and health, and views people as occupational beings [1]. “Occupational therapists should continue to be mindful of the humanistic ideals on which the profession was founded: the belief in the therapeutic value of meaningful occupation, the importance of the environment and of satisfactory interpersonal relationships, and balance in the daily routines of work, self-care and leisure” [13].

In Germany in particular, OT in inpatient psychiatric settings is mainly performed in group settings and is composed of four therapeutic facets. The first facet is the classical *Ergotherapie* that is the performance of various handcraft techniques with wood, stone, paper, and other materials. The second facet addresses the expression of inner states through drawing, painting, or modeling. The third facet focuses on the interactions and social skills of the group members while they are involved in common projects. The fourth facet concentrates on work performance and workplace reintegration aspects.

The extensive provision of OT for psychiatric patients in German-speaking countries causes marked costs, particularly in inpatient settings. According to Reuster [14], OT for inpatients in the Department of Psychiatry and Psychotherapy of the University of Dresden in 1998 was more expensive than pharmacotherapy for those patients. However, empirical data regarding the effectiveness and efficacy of OT in patients with mental disorders are lacking, and there are only few randomized controlled trials (RCTs) in this field.

Comparatively strong evidence exists for OT in community samples of people with dementia. Voigt-Radloff et al. [17] stated that the results of 5 of 7 RCTs suggested positive effects on activities of daily living (ADLs) or quality of life in persons with dementia or on their caregivers’ skills, burden, and quality of life.

To date, OT for patients with schizophrenia has been investigated only in long-term RCTs [3, 4, 6].

Schene et al. [15] were the first to perform a long-term RCT on OT (vs. treatment as usual, TAU) in outpatients with major depressive disorder; however, in that study, the OT intervention was not superior to TAU with respect to depression outcome. In a subsequent similar RCT (TAU + OT vs. TAU) in sick-listed employees with major depression, the workgroup focused on *work participation* as the primary outcome parameter, but significant benefits of adjuvant OT pertaining to a quick return to work, improvement of work-related coping and self-efficacy were not demonstrated. However, the OT group showed greater improvement in depression symptoms and an increased probability of long-term symptom remission and long-term *return to work in good health* [9].

Reuster [14] was the first to conduct a short-term RCT in 216 inpatients with major depression (*n* = 114; n_+OT_ = 63, *n*_-OT_ = 51), mania (*n* = 26; *n*_+OT_ = 16, *n*_−OT_ = 10), and schizophrenia (*n* = 76; *n*_+OT_ = 41, *n*_−OT_ = 35). That author investigated the effects of daily add-on standard OT (performance and training of handcraft techniques using wood, stone, paper, and other materials) versus self-instructed unspecific activities on psychopathological variables over four weeks within a multimodal clinical setting. A significant reduction of symptomatology was only observed in the patients with major depression (43.9% decrease in the Bech Rafaelsen Melancholia Scale score vs. a 27.5% decrease in the activity group after 4 weeks). However, that study had multiple methodological problems; it lacked a primary outcome parameter, an assessment of functioning in ADL, and control for confounders, such as drug therapy, psychoeducative and psychotherapeutic sessions, and other covariates, such as exercise therapy.

Because the above-mentioned study by Reuster is the only investigation of short-term OT in inpatients with major depression to date, we felt inspired to study this topic further.

We planned and performed a pilot RCT in inpatients with moderate-to-severe major depression that compared a standard OT group program, i.e., everyday performance of handcraft activities, to a board game (BG) group as a semi-active control. In both groups, the interventions were in addition to basic antidepressant drug treatment and short daily supportive talks with staff members.

We avoided the major limitations of Reuster’s study by defining the change in the total score of the Hamilton Depression Rating Scale (HAMD) from before to after the interventions as the primary outcome parameter. Moreover, we applied secondary assessments of psychopathological symptoms, such as anxiety, and we included a specific OT assessment (Ergo-Assess™) of functioning in ADLs, and we controlled for confounders (antidepressant drugs and psychiatric comorbidity).

We expected that the adjuvant OT intervention would result in significantly greater effects indicating decrease in depressive symptoms and secondary psychopathological characteristics. Furthermore, we hypothesized superiority of the OT over the BG intervention with respect to improvements in ADL and social functioning. Additionally, gender differences regarding effects indicating possible improvements were explored.

## Methods

### Participants

A total of 131 inpatients who experiences a moderate or severe major depressive episode diagnosed according to the DSM-IV criteria (moderate, 296.22, or severe episode without psychotic features, 296.23; recurrent major depressive disorder: moderate, 296.32, or severe episode without psychotic features, 296.33) were recruited from three similar inpatient units of two German psychiatric clinics and assessed for eligibility for participation in this study. All diagnoses were established using the Structured Clinical Interview for the DSM-IV (SCID I/II). Of the 131 patients who were screened, 14 did not meet the inclusion criteria and three refused to participate. Finally, 114 patients (55% female, mean age 45.7 ± 11.8 years) were randomly (by the block random method) assigned to either the experimental (OT) or the active comparison group (board game group, BG). During the first 3 weeks, 11 (19.3%) OT participants and 21 (36.8%) BG participants dropped out for motivational reasons. 46 OT participants and 36 BG participants participated in the study for at least three weeks (*n* = 82) and 29 OT participants and 22 BG participants completed the study (6 weeks, *n* = 51). The data were processed in per-protocol analyses.

No significant group differences emerged regarding sex, age, education, marital status, intelligence, axis-I or axis-II comorbidity, number of psychoactive drugs, or number of antidepressants. However, as the only particular difference between study groups, the female participants in the OT group took significantly more psychoactive drugs (*p* = 0.020) than the female participants in the BG group (Table 1). No overall gender differences were found between study groups concerning intelligence, axis-I or axis-II comorbidity, number of psychoactive drugs, or number of antidepressants.

**Table 1:** Group comparisons (demographic and clinical data)

	Occupational therapy	Board game group	Chi square^a^/t^b^	df	*p*
Demographic data					
*N* = 82	*n* = 46	*n* = 36			
Sex (f:m)	26:20	18:18	0.38^a^	1	0.539
Age, years (SD)	46.8 (11.8)	44.8 (11.7)	0.84^b^	81.8	0.401
Age, females (SD)	45.7 (10.7)	45.4 (13.3)	0.11^b^	40.4	0.910
Age, males (SD)	48.2 (13.3)	44.1 (9.8)	1.18^b^	31.5	0.247
Age groups	6 groups ranging from 18 to 70 years of age		2.74^a^	5	0.740
Educational level	11 categories ranging from ‘no degree’ to ‘university degree’		17.3^a^	10	0.068
Marital status	4 categories (single, married, divorced, widowed)		577^a^	3	0.124
Clinical data					
Intelligence, MWT-B, raw data (SD)	29.1 (14.8)	28.1 (4.8)	0.50^b^	71.2	0.617
Intelligence, females (SD)	30.3 (9.0)	27.7 (4.7)	0.77^b^	37.7	0.448
Intelligence, males (SD)	27.5 (4.8)	28.5 (5.0)	−0.70^b^	37	0.487
Axis-I disorders (SD)	1.7 (0.8)	1.5 (0.7)	0.18^b^	75	0.861
Axis-I disorders, females (SD)	1.8 (0.9)	1.4 (0.6)	1.62^b^	35.8	0.112
Axis-I disorders, males (SD)	1.3 (0.6)	1.7 (0.8)	−1.74^b^	36	0.090
Axis-II disorders (SD)	0.2 (0.4)	0.1 (0.3)	0.60^b^	75	0.550
Axis-II disorders, females (SD)	0.2 (0.4)	0.1 (0.3)	0.22^b^	39.7	0.827
Axis-II disorders, males (SD)	0.2 (0.4)	0.1 (0.3)	0.64^b^	36	0.524
Number of psychoactive drugs (SD)	2.6 (1.4)	2.4 (1.3)	0.63^b^	72	0.529
Number of psychoactive drugs, females (SD)	2.6 (1.3)	2.0 (0.8)	2.40^b^	42	0.020
Number of psychoactive drugs, males (SD)	2.4 (1.4)	2.9 (1.8)	−0.99^b^	36	0.331
Number of antidepressants (SD)	1.8 (1.0)	1.7 (0.8)	0.20^b^	77.8	0.848
Number of antidepressants, females (SD)	1.8 (0.8)	1.6 (0.8)	1.02^b^	38.7	0.311
Number of antidepressants, males (SD)	1.7 (1.2)	1.9 (0.72)	−0.64^b^	36	0.524

^a^ relates to Pearson's Chi-square tests

^b^ relates to *t* tests

Over 80% of the participants took at least one antidepressant drug before admission. Of these participants, only about 20% had only one antidepressant.

All participants gave written informed consent, and the Ethics Committee of the Medical Faculty of the Ruhr University Bochum approved the study (No. 3626-10FF).

### Study design, interventions, and inclusion/exclusion criteria

The study was primarily designed as a 6-week pilot RCT with a block randomization. However, block size could not be fixed randomly in this study, but the trialists allocated blocks of three, four, or five participants to the groups alternately, according to the availability of eligible patients.

Basic handcraft is the core OT activity in German-speaking countries. Therefore, the primary or experimental intervention comprised standardized performance of basic handcraft activities, such as painting and crafting with wood, stone, and other materials.

Board game activities were used for control condition. The resemblance of such activities with OT in a stricter sense should improve the acceptability of the control intervention, since many patients claim OT as an essential part of inpatient treatments.

Both interventions were conducted 2 h daily, 5 days a week. Only one handcraft activity (either crafting or painting) or board game (like Monopoly or cards, involving more than two persons, thus no chess or Scrabble) was performed in each 2-h session. Both interventions were provided for groups with 6–8 patients and conducted by professional occupational therapists. No cognitive or talk therapy was added to the interventions, but a basic antidepressant drug treatment and supportive or psychoeducative talks up to 20 min per day with staff members were allowed.

The inclusion criteria were the following: 18- to 65-year-old inpatients with moderate or severe major depression without psychotic or catatonic features (moderate, 296.22, or severe episode without psychotic features, 296.23; recurrent major depressive disorder: moderate, 296.32, or severe episode without psychotic features, 296.33) and a Hamilton Depression Rating Scale (HAMD-21) score ≥18. Any antidepressant medication was allowed, if possible as a mono-therapy. Z-drugs were permitted to treat sleep problems. In case of restlessness or agitation, promethazine (up to 75 mg per day), lorazepam (up to 3 × 1 mg per day) or quetiapine (up to 100 mg per day) could be prescribed.

The exclusion criteria were the following: contraindications for antidepressants; currently at risk of suicide; and a DSM-IV diagnosis of any of the following: dementia, schizophrenia spectrum disorders, cluster A and cluster B personality disorders, substance use disorders (abuse and dependence), eating disorders (anorexia and bulimia nervosa); acute, serious, or unstable medical conditions; and pregnancy in females.

### Outcome measures

The primary outcome parameter was *decrease in depressivity* as measured by the Hamilton Depression Rating Scale, HAMD-21 [7]. The Beck Depression Inventory, BDI [2], was used as a secondary outcome measure. Compared to the (interviewer-rating) HAMD, the (self-rating) BDI assesses rather subjective depressivity, and reductions of BDI scores during therapy may depend on personality traits, particularly introversion and neuroticism, to a larger extent, than do changes in HAMD scores. Therefore, complementary performance of both instruments may be useful [16]. State anxiety was measured using the Hamilton Anxiety Rating Scale, HAMA [8]. Furthermore, the Personal and Social Performance Scale, PSP, an interviewer-rating instrument, was used to assess four features of social functioning (socially useful activities; personal and social relationships; self-care; and disturbing and aggressive behavior) over a one-month period [12, 11]. These scales were applied for screening and baseline ratings and for the follow-up assessments at 3–9 weeks after baseline. A physician and a psychologist from our work group carried out the assessments. The software package *Ergo*-*Assess™* [10] was used to assess functioning in activities of daily living (ADL) in the six domains of the International Classification of Functioning, Disability and Health, ICF [19]: (1) activities of physical self-care, (2) activities of independent living, (3) neuropsychological functioning, (4) psychosocial functioning, (5) sensomotoric functions, and (6) basic work activities. In contrast to the other instruments, Ergo-Assess was used at 1–6 weeks, as opposed to 3–6 weeks, after baseline by a professional occupational therapist.

### Data analysis

Statistical analyses were carried out using the Statistical package for the Social Sciences (SPSS™), version 20 for Mac, IBM Corp., Armonk, New York, United States. The one-sample Kolmogorov–Smirnov test was used to confirm that all interval-scaled variables were normally distributed. Group comparisons in respect to gender, age, education, depressive symptomatology, comorbidity, and medication were conducted using *t* tests and Pearson’s Chi-square tests. A reduction of the HAMD-21 total score of ≥50% from baseline was defined as ‘antidepressive response,’ and a HAMD-21 total score of ≤7 was defined as *remission*. The groups were compared with respect to these HAMD factors by performing Pearson’s Chi-square tests. Possible treatment effects were investigated using general linear models (GLM) with repeated measures analyses of variance. Age, IQ, axis-I and axis-II comorbidity, number of psychoactive drugs, and number of antidepressants were taken into account as covariates. Cohen’s measure of sample effect size for comparing two samples means, i.e., pre- and post-means, was then used to assess possible treatment effects. Results with *p* < 0.05 were considered statistically significant.

## Results

### Antidepressive response and remission

No significant group differences were found in terms of antidepressive response or remission after three- and six-week treatment. Antidepressive response was found in 10 participants (21.7%) of the OT group (*n* = 46) and 13 participants (36.1%) of the BG group (*n* = 36) after 3 weeks, and in 19 participants (65.5%) of the OT group (*n* = 29) and 12 participants (54.5%) of the BG group (*n* = 22) after 6 weeks. Remission was found in nine participants (19.6%) in the OT group vs. five participants (13.9%) in the BG group (Chi-square = 0.536, *df* = 1, *p* = 0.464) after 3 weeks, and in eight participants (27.6%) in the OT group vs. nine participants (40.9%) in the BG group after 6 weeks (Chi-square = 0.777, *df* = 1, *p* = 0.378).

### Primary outcome parameter

The GLM analysis did not find any significant time-by-group interaction effects regarding the primary outcome parameter *HAMD total score* (after 3 weeks: *F* = 0.141, *p* = 0.709; after 6 weeks: *F* = 0.177, *p* = 0.828). This indicates that neither group reached antidepressive superiority.

### Secondary outcome parameters

A significant time-by-group interaction effect regarding the HAMA total score in males after three weeks was observed which suggests superiority of the OT intervention over the BG intervention (*F* = 5.226, *p* = 0.031; *d* = 1.23 vs. 0.48) (Table 2). At 6 weeks, no significant interaction effect was found. No other significant interaction effects were observed regarding the other total scores (BDI, PSP and ErgoAssess).

**Table 2:** Occupational therapy (*n* = 46) vs. board games (*n* = 36): general linear model (significant interaction effects at baseline and after 3 weeks)

Variable	Subgroup	GLM						Means, differences (pre-post) and effect sizes							
		Within-subject effects (time)		Between-subjects effects (group)		Interaction (time × group)		Occupational therapy				Board game group			
		F	*p*	F	*p*	F	*p*	M pre (SD)	M post (SD)	∆_Mpre-Mpost_	ES	M pre (SD)	M post (SD)	∆_Mpre-Mpost_	ES
BDI 12	Total	0.429	0.515	4.579	0.036	1.001	0.298	1.21 (0.87)	0.83 (0.74)	0.38	0.47	0.83 (0.75)	0.65 (0.65)	0.18	0.26
	m	0.010	0.922	1.237	0.276	13.494	0.001	1.25 (0.90)	0.53 (0.61)	0.72	0.95	0.53 (0.70)	0.81 (0.75)	–	–
	f	0.000	0.992	2.445	0.128	2.559	0.119	1.18 (0.86)	1.04 (0.76)	0.14	0.16	1.10 (0.70)	0.50 (0.51)	0.60	0.98
BDI 16	Total	0.003	0.954	0.769	0.376	4.983	0.029	1.63 (1.09)	1.30 (1.07)	0.33	0.31	1.90 (1.01)	1.06 (0.81)	0.84	0.92
	m	0.000	0.991	3.057	0.092	1.047	0.315	1.50 (1.02)	1.16 (1.07)	0.34	0.32	2.00 (1.08)	1.13 (0.72)	0.87	0.97
	f	0.006	0.937	0.006	0.938	1.607	0.214	1.75 (1.14)	1.41 (1.08)	0.34	0.31	1.82 (0.96)	1.00 (0.91)	0.82	0.87
BDI 22	Total	0.481	0.490	0.000	0.993	3.094	0.083	1.52 (1.16)	1.33 (1.12)	0.19	0.17	1.37 (1.20)	1.47 (1.16)	–	–
	m	2.190	0.150	0.493	0.489	5.017	0.034	1.54 (1.06)	1.00 (1.00)	0.54	0.52	1.30 (1.13)	1.55 (1.13)	–	–
	f	0.022	0.883	0.271	0.606	0.150	0.702	1.50 (1.26)	1.56 (1.16)	–	–	1.43 (1.29)	1.55 (1.29)	–	–
HAMA total	Total	0.635	0.428	5.715	0.020	1.291	0.260	20.07 (7.06)	12.86 (6.73)	7.21	1.04	20.95 (6.75)	16.60 (8.78)	4.35	0.56
	m	0.023	0.882	4.511	0.044	5.226	0.031	19.38 (7.56)	10.94 (5.07)	8.52	1.23	19.11 (5.61)	16.35 (5.78)	2.76	0.48
	f	0.108	0.744	2.060	0.160	0.120	0.731	20.58 (6.75)	14.12 (7.45)	6.46	0.91	22.55 (7.34)	16.83 (11.07)	5.72	0.62
HAMA 6	Total	0.230	0.633	3.001	0.088	4.190	0.044	2.81 (0.64)	1.84 (0.97)	0.97	1.20	2.83 (0.71)	2.23 (0.81)	0.60	0.79
	m	0.023	0.880	0.305	0.586	3.669	0.067	2.88 (0.45)	1.88 (0.86)	1.00	1.52	2.74 (0.56)	2.24 (0.66)	0.50	0.82
	f	0.010	0.919	2.156	0.151	0.189	0.666	2.76 (0.75)	1.81 (1.06)	0.95	1.04	2.91 (0.87)	2.22 (0.94)	0.69	0.77
PSP C	Total	0.000	0.991	1.309	0.256	1.908	0.171	1.20 (0.56)	1.16 (0.42)	0.04	0.08	1.36 (0.62)	1.17 (0.51)	0.19	0.33
	m	0.835	0.368	0.283	0.599	0.013	0.911	1.33 (0.76)	1.21 (0.54)	0.12	0.18	1.35 (0.59)	1.22 (0.55)	0.13	0.36
	f	1.400	0.245	3.341	0.076	5.213	0.029	1.10 (0.30)	1.12 (0.33)	–	–	1.36 (0.66)	1.11 (0.47)	0.25	0.44

BDI 12: loss of interest

BDI 16: disturbed sleep pattern

BDI 22: loss of sexual interest

HAMA_total_: total score

HAMA 6: depressed mood

PSP C: self-care

*GLM* general linear model (controlled for age, IQ, axis-I/II comorbidity, number of psychoactive drugs and number of antidepressants), stratified for gender (*m* males, *f* females); *M pre/post* mean pre/post, *SD* standard deviation, *∆*_*Mpre*-*Mpost*_ difference between re- and post-mean, *ES* effect size (Cohen’s d). *BDI* Beck Depression Inventory, *HAMA* Hamilton Anxiety Scale, *PSP* Personal and Social Performance Scale

### Subscale parameters

#### Comparison after 3 weeks

The following significant time-by-group interactions were observed: *loss of interest* (BDI 12) in favor of the OT group (*F* = 13.494, *p* = 0.001; *d* = 0.95 vs. 0.00) in the male participants; *disturbed sleep pattern* (BDI 16) in favor of the BG group (*F* = 4.983, *p* = 0.029; *d* = 0.92 vs. 0.31) in both genders; *loss of sexual interest* (BDI 22) in favor of the OT group in the male participants (*F* = 5.017, *p* = 0.034; *d* = 0.22 vs. 0.00); *depressed mood* (HAMA 6) in favor of the OT group in both genders (*F* = 4.190, *p* = 0.044; *d* = 1.20 vs. 0.79); and *self*-*care* (PSP C) in favor of the BG group in the female participants (*F* = 5.213, *p* = 0.029; *d* = 0.44 vs. 0.00) (Table 2).

#### Comparison after 6 weeks

The following significant time-by-group interaction effects were found subscales of the various inventories assessed: *depersonalization and derealization* (HAMD 19) in favor of the BG group in all participants (*F* = 4.321, *p* = 0.044; *d* = 0.71 vs. 0.00) and in the subgroup of male participants (*F* = 4.944, *p* = 0.039; *d* = 0.83 vs. 0.00); *loss of energy* (BDI 15) in favor of the OT group in all participants (*F* = 5.095, *p* = 0.030; *d* = 1.05 vs. 0.46); *disturbed sleep pattern* (BDI 16) in favor of the OT group in the subgroup of female participants (*F* = 6.415, *p* = 0.025; *d* = 0.92 vs. 0.51); *loss of sexual interest* (BDI 22) in favor of the OT group in all participants (*F* = 11.908, *p* = 0.001; *d* = 0.36 vs. 0.00) and in the subgroup of male participants (*F* = 6.642, *p* = 0.028; *d* = 0.57 vs. 0.00); *anxious behavior during the interview* (HAMA 14) in favor of the OT group in the subgroup of male participants (*F* = 6.301, *p* = 0.022; *d* = 1.26 vs. 0.41); and *basic work skills* (ErgoAssess 3) in favor of the OT group in all participants (*F* = 6.344, *p* = 0.017; *d* = 1.83 vs. 0.16) (Table 3).

**Table 3:** Occupational therapy (*n* = 29) vs. board games (*n* = 22): general linear model (significant interaction effects at baseline and after 6 weeks)

Variable	Sub-group	GLM						Means, differences (pre-post) and effect sizes							
		Within-subject effects (time)		Between-subjects effects (group)		Interaction (time × group)		Occupational therapy				Board game group			
		F	*p*	F	*p*	F	*p*	M pre (SD)	M post (SD)	∆_Mpre-Mpost_	ES	M pre (SD)	M post (SD)	∆_Mpre-Mpost_	ES
HAMD 19	Total	3.682	0.062	2.500	0.121	4.321	0.044	0.02 (0.13)	0.03 (0.19)	–	–	0.12 (0.33)	0.00 (0.00)	0.12	0.71
	m	1.814	0.194	3.291	0.085	4.944	0.039	0.00 (0.00)	0.06 (0.25)	–	–	0.10 (0.31)	0.00 (0.00)	0.10	0.63
	f	1.003	0.331	2.509	0.132	2.509	0.132	0.03 (0.17)	0.00 (0.00)	0.03	0.33	0.14 (0.35)	0.00 (0.00)	0.14	0.78
BDI 15	Total	0.290	0.594	0.060	0.808	5.095	0.030	1.88 (0.70)	1.11 (0.75)	0.77	1.05	1.67 (0.85)	1.25 (0.97)	0.42	0.46
	m	1.452	0.245	0.202	0.659	0.928	0.349	1.88 (0.68)	1.06 (0.77)	0.82	1.12	1.65 (0.75)	1.11 (0.93)	0.56	0.67
	f	0.025	0.877	0.850	0.373	0.757	0.400	1.89 (0.74)	1.18 (0.75)	0.71	0.95	1.68 (0.95)	1.36 (1.03)	0.32	0.32
BDI 16	Total	0.008	0.930	7.164	0.011	0.555	0.461	1.63 (1.09)	0.81 (0.74)	0.82	0.89	1.90 (1.01)	1.43 (1.17)	0.47	0.43
	m	1.205	0.287	7.065	0.016	0.075	0.787	1.50 (1.02)	0.81 (0.66)	0.69	0.82	2.00 (1.08)	1.60 (1.17)	0.40	0.35
	f	6.150	0.028	0.002	0.970	6.415	0.025	1.75 (1.14)	0.82 (0.87)	0.93	0.92	1.82 (0.96)	1.27 (1.19)	0.55	0.51
BDI 22	Total	0.002	0.960	0.156	0.695	11.908	0.001	1.52 (1.16)	1.11 (1.12)	0.41	0.36	1.37 (1.20)	1.55 (1.18)	–	–
	m	0.023	0.882	0.313	0.583	5.642	0.028	1.54 (1.06)	0.94 (0.93)	0.60	0.57	1.30 (1.13)	1.55 (1.13)	–	–
	f	0.526	0.481	0.775	0.395	0.114	0.741	1.50 (1.26)	1.36 (1.36)	0.14	0.11	1.43 (1.29)	1.55 (1.29)	–	–
HAMA 14	Total	0.131	0.720	0.779	0.283	2.412	0.128	1.19 (0.93)	0.38 (0.62)	0.81	1.04	1.07 (0.69)	1.55 (4.64)	–	–
	m	2.839	0.109	0.489	0.493	6.301	0.022	1.29 (1.12)	0.38 (0.62)	0.91	1.26	0.95 (0.71)	0.64 (0.81)	0.31	0.41
	f	0.101	0.755	1.332	0.265	1.424	0.250	1.12 (0.78)	0.38 (0.65)	0.74	1.03	1.18 (0.66)	2.45 (6.53)	–	–
Ergo-Asses 3	Total	1.753	0.195	0.172	0.681	6.344	0.017	13.90 (3.51)	10.96 (3.59)	2.94	0.83	12.72 (5.12)	11.90 (5.03)	0.82	0.16
	m	1.036	0.329	3.697	0.079	2.245	0.160	14.45 (4.12)	11.92 (4.38)	2.53	0.60	11.94 (5.49)	9.70 (3.53)	2.24	0.50
	f	0.667	0.429	3.089	0.102	2.210	0.161	13.50 (3.01)	9.91 (2.21)	3.59	1.38	13.38 (4.81)	14.10 (5.49)	–	–

HAMD 19: depersonalization and derealization

BDI 15: loss of energy

BDI 16: disturbed sleep pattern

BDI 22: loss of sexual interest

HAMA 14: anxious behavior during interview

Ergo-Assess 3: basic work skills

*GLM* general linear model (controlled for age, IQ, axis-I/II comorbidity, number of psychoactive drugs and number of antidepressants), stratified for gender (*m* males, *f* females), *M pre/post* mean pre/post, *SD* standard deviation, *∆*_*Mpre*-*Mpost*_ difference between pre- and post-mean, *ES* effect size (Cohen’s d). *HAMD* Hamilton Depression Scale, *BDI* Beck Depression Inventory, *HAMA* Hamilton Anxiety Scale, *Ergo*-*Assess™* German Occupational Therapy Assessment

## Discussion

Data on adjuvant occupational therapy (OT) in inpatient psychiatric settings is greatly lacking. In fact, there have only been two studies on this topic [6, 14] to date. Only one trial by Reuster evaluated the effects of short-term adjuvant OT; that study was on inpatients with schizophrenia, mania, and major depression. Unfortunately, that study has not been published in PubMed, and it is the only available work that is comparable to ours.

In this study, our purpose was to investigate the effects of short-term adjuvant OT in patients with a mental disorder of considerable epidemiologic and clinical relevance, i.e., major depression, in an inpatient (i.e., costly) setting. In German-speaking countries, OT is broadly applied and generally financed by health insurance companies. The main difficulty in designing this study was that a simple comparison of pharmacotherapy alone (preferably with a single drug) to pharmacotherapy plus OT was not possible because the standard psychiatric inpatient settings in German-speaking countries provide pharmacotherapy, psycho-education and psychotherapy in single and group settings and exercise therapy, different occupational therapies, and other treatments. Therefore, we attempted to merge the demands of patients and the requirements of health insurance companies into a study design that was as simple as possible and, most importantly, had the least possible number of confounders.

Our main finding was that the interventional OT group was not superior to the control board game (BG) group with respect to our primary outcome measures: The study did not show any reduction of depressivity and percentage of remissions as measured by the Hamilton Depression Scale (HAMD-21). However, some significant time-by-group effects indicated a superiority of the OT intervention over the BG intervention with respect to anxiety, i.e., reductions in anxiety in general (HAMA total score) in male participants after three weeks, depressed mood (HAMA subscale) in participants of both genders after three weeks, and anxious behavior during the interview (HAMA subscale) in male participants after six weeks.

Moreover, significant interaction effects in regards to some subscales of the Beck Depression Inventory (BDI) indicated the superiority of OT over BG, including loss of general interest and loss of sexual interest in males after 3 weeks, loss of energy in all participants after six weeks, disturbed sleep pattern in females after 6 weeks, and loss of sexual interest in both genders after 6 weeks. However, a significant interaction effect with respect to disturbed sleep pattern in participants of both genders after 3 weeks suggested a superiority of BG over OT. Other measures in favor of BT included self-care (from the Personal and Social Performance Scale) after 3 weeks in the female participants and depersonalization and derealization (from the HAMD) after 6 weeks in participants of both genders.

Finally, basic work skills (assessed with Ergo-Assess™) improved significantly more in the OT participants of both genders after 6 weeks. Effect sizes with respect to the superior group, i.e., predominantly the OT group, were mainly in the high range (*d* > 0.8).

There were several limitations to this study: We faced difficulties concerning the comparability of the two groups; for example, patients found the OT intervention much more pleasant and effective than the BG activities, which was reflected by a far greater dropout rate during the first 3 weeks in the BG group, 36.8%, compared to the OT group, 19.3%.

The (“semi-active”) BG group was not a true control group, such as a placebo or waitlist group; thus, we can only discuss ‘effects’ but not ‘efficacy’ of the OT intervention in comparison to the BG activities.

For comparability reasons, each group intervention was completely structured and standardized as to content and procedure, which entailed limited performance of individually meaningful activities and accomplishment of personal goals. Thus, the OT intervention was limitedly representative of standard OT group interventions for inpatients. Moreover, our “OT” intervention is by no means representative of occupational therapy in general.

We were not able to explain why BDI but not HAMD scores decreased during the intervention. Changes in BDI scores rather than HAMD scores emerged to be associated with personality features like introversion and neuroticism [16]; however, this study lacked assessment of personality traits.

Insufficient qualitative and process-related assessment of pharmacotherapy represents a major shortcoming of this study. However, the numbers of psychoactive and antidepressant drugs were considered as covariates, and 80% of the participants were known to enter the study taking at least one antidepressant.

Finally, the study was underpowered, as it had an even smaller study sample size than that of Reuster’s investigation (82 participants in our study vs. 114 participants in Reuster’s study). Thus, this study has to be regarded as a pilot project.

## Conclusions

In conclusion, the OT group in our study showed more significant effects indicating improvement with respect to features of anxiety, loss of energy, sexual and general interest, and work skills than the BG group. Moreover, the results of this study suggest a greater benefit of the OT intervention in males than in females.

Together, our results suggest that adjuvant standard occupational treatment may be superior to mere board game activities and may be a feasible adjunct therapy to pharmacotherapy (and possibly other treatments) in a psychiatric short-term setting for inpatients with major depression.

## Abbreviations

ADL: activities of daily living
BDI: Beck Depression Inventory
BG (group): board game (group)
DSM-IV: Fourth edition of the Diagnostic and Statistical Manual of the American Psychiatric Association
Ergo-Assess™: instrument for the assessment of occupational therapy outcome
GLM: general linear model
HAMA: Hamilton Anxiety Rating Scale
HAMD (-21): Hamilton Depression Rating Scale (with 21 items)
ICF: International Classification of Functioning, Disability and Health
OT (group): occupational therapy (group)
PSP: scale personal and social performance scale
RCT: randomized controlled trial
SCID: Structured Clinical Interview for the DSM-IV
SPSS: statistical package for the social sciences
TAU: treatment as usual
WHO: World Health Organization
